# PGC-1*α*, Inflammation, and Oxidative Stress: An Integrative View in Metabolism

**DOI:** 10.1155/2020/1452696

**Published:** 2020-03-09

**Authors:** Sergio Rius-Pérez, Isabel Torres-Cuevas, Iván Millán, Ángel L. Ortega, Salvador Pérez

**Affiliations:** ^1^Department of Physiology, Faculty of Pharmacy, University of Valencia, 46100 Burjassot, Valencia, Spain; ^2^Neonatal Research Group, Health Research Institute La Fe, 46026 Valencia, Spain

## Abstract

Peroxisome proliferator-activated receptor-*γ* coactivator (PGC)-1*α* is a transcriptional coactivator described as a master regulator of mitochondrial biogenesis and function, including oxidative phosphorylation and reactive oxygen species detoxification. PGC-1*α* is highly expressed in tissues with high energy demands, and it is clearly associated with the pathogenesis of metabolic syndrome and its principal complications including obesity, type 2 diabetes mellitus, cardiovascular disease, and hepatic steatosis. We herein review the molecular pathways regulated by PGC-1*α*, which connect oxidative stress and mitochondrial metabolism with inflammatory response and metabolic syndrome. PGC-1*α* regulates the expression of mitochondrial antioxidant genes, including manganese superoxide dismutase, catalase, peroxiredoxin 3 and 5, uncoupling protein 2, thioredoxin 2, and thioredoxin reductase and thus prevents oxidative injury and mitochondrial dysfunction. Dysregulation of PGC-1*α* alters redox homeostasis in cells and exacerbates inflammatory response, which is commonly accompanied by metabolic disturbances. During inflammation, low levels of PGC-1*α* downregulate mitochondrial antioxidant gene expression, induce oxidative stress, and promote nuclear factor kappa B activation. In metabolic syndrome, which is characterized by a chronic low grade of inflammation, PGC-1*α* dysregulation modifies the metabolic properties of tissues by altering mitochondrial function and promoting reactive oxygen species accumulation. In conclusion, PGC-1*α* acts as an essential node connecting metabolic regulation, redox control, and inflammatory pathways, and it is an interesting therapeutic target that may have significant benefits for a number of metabolic diseases.

## 1. Introduction

Peroxisome proliferator-activated receptor-*γ* coactivator (PGC)-1*α* is a transcriptional coactivator that was initially identified in an interaction with nuclear receptor peroxisome proliferator-activated receptors (PPAR*γ*) in response to lower temperatures [1]. In addition to its role in adaptive thermogenesis, PGC-1*α* is presently described as a master regulator of mitochondrial biogenesis and function, including oxidative phosphorylation (OXPHOS) and reactive oxygen species (ROS) detoxification [2]. In recent years, PGC-1*α* has been associated with many inflammatory and metabolic diseases, and its crucial role regulating mitochondrial function, oxidative stress, and metabolic pathways in diverse tissues has been revealed [3–6]. We herein review the different functions and molecular pathways regulated by PGC-1*α*, which connect oxidative stress and mitochondrial metabolism with inflammatory response and metabolic syndrome.

## 2. PGC-1*α*

### 2.1. PGC-1 Family of Transcriptional Coactivators

The PGC-1 family consists of three members, namely, PGC-1*α*, PGC-1*β*, and PGC-related coactivator (PRC), which interact with a whole range of transcription factors involved in a wide variety of biological functions [7]. As a result of these interactions, the transcriptional activity of these factors and the biological response associated with them end up being modulated by PGC-1*α* [7, 8].

**Figure 1:**
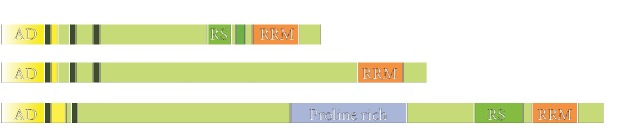
Structure of PGC-1 family coactivators.

PGC-1 family members exhibit a high degree of amino acid sequence homology, especially in amino- and carboxy-terminal regions (Figure 1) [8]. The amino-terminal region of all PGC-1 coactivators contains a highly conserved activation domain required for the recruitment of histone acetyltransferase proteins steroid receptor coactivator-1 (SRC-1) and cAMP response element-binding (CREB) binding protein (CBP)/p300 which, in turn, favors the access of the transcriptional complex to DNA [9]. The N-terminal domain also contains several leucine-rich LXXLL motifs (NR boxes) that are crucial for the interaction between PGC-1 and their transcriptional partners [1, 10]. The carboxy-terminal region contains a well-conserved RNA recognition motif (RRM), which has been recognized to be involved in both RNA and single-stranded DNA binding [8, 11]. Additionally, RS domains (short serine/arginine-rich stretches) are located in the N-terminal to the RRM motif in PGC-1*α* and PRC, but not in PGC-1*β* [12, 13]. Interestingly, the RS and RRM motifs are typically found in proteins involved in RNA splicing, which suggests that PGC-1 coactivators interact with splicing machinery [2, 8, 14].

Although the expression pattern of PGC-1*α* and PGC-1*β* is similar, PGC-1*α* exhibits considerable versatility for being expressed in different physiological situations, which require high energy expenditure [8]. In fact, PGC-1*α* is highly expressed in tissues with active oxidative metabolism, such as brown adipose tissue (BAT), heart, skeletal muscle, and brain, but is expressed at low levels in white adipose tissue (WAT) [7, 11]. In this review, we focus on the role that PGC-1*α* plays in inflammatory response, which is commonly accompanied by energy expenditure and metabolic disturbances.

### 2.2. Regulation of PGC-1*α*

PGC-1*α* is regulated at both the transcriptional and post-translational levels [15]. Different nutritional and environmental stimuli associated with energy stress, including exercise, cold exposure, or fasting, induce PGC-1*α* expression in different cell types [11]. CREB, myocyte enhancer factor 2 (MEF2), activating transcription factor 2 (ATF2), forkhead Box O1 (FoxO1), and forkhead box O3A (FoxOA3) are the most important transcription factors that control *PGC-1α* gene expression in a tissue-dependent manner [15].

The transcriptional regulation of PGC-1*α* is orchestrated mainly by CREB activation in different tissues [11]. The *PGC-1α* gene exhibits a well-conserved binding site for CREB, which drives PGC-1*α* expression after its activation [16]. In skeletal muscle cells, intracellular calcium levels increase in response to exercise, which induces calcium/calmodulin-dependent protein kinase IV (CaMKIV)—dependent phosphorylation and the subsequent activation of CREB [17–19]. In BAT and muscle cells, cold temperature stimulates cAMP signaling and protein kinase A (PKA), which promotes the downstream activation of CREB [20]. Likewise, glucagon-dependent cAMP and CREB activation triggers PGC-1*α* expression in the liver during fasting [16]. In many cell types, the p38 mitogen-activated protein kinase (MAPK) signaling pathway is simultaneously activated with CREB to upregulate *PGC-1α* gene expression. p38 MAPK can induce PGC-1*α* expression by activating both MEF2 and ATF2 [20, 21]. In BAT, *β*3-adrenergic receptor and cAMP/PKA activation by cold temperature stimulate the p38 MAPK signaling pathway, which in turn triggers *PGC-1α* gene expression through ATF2 [20]. Similarly in the fasting liver, cAMP-PKA axis promotes the activation of PGC-1*α* by p38 MAPK [22]. FoxO transcription factors also contribute to the transcriptional regulation of PGC-1*α* in different cell types. Inactivation of FoxOA3 by the phosphatidylinositol-4,5-bisphosphate 3-kinase-serine/threonine protein kinase B (PI3K/Akt) signaling pathway promotes PGC-1*α* downregulation in endothelial cells [23]. PGC-1*α* expression is partially controlled by FoxO1 in HepG2 cells, but this regulation is inhibited in response to insulin through Akt-dependent FoxO1 phosphorylation [24]. Furthermore, epigenetic modifications in the promoter of the *PGC-1α* gene are now emerging as novel mechanisms to regulate PGC-1*α* expression. In muscle cells, *PGC-1α promoter methylation by* DNA methyltransferase 3B (DNMT3B) represses *PGC-1α* expression in response to high levels of fatty acids [25]. The promoter region of *PGC-1α* is enriched in lysine-specific demethylase-1 (LSD1) in adipocytes, where *PGC-1α* is not expressed [26]. In these cells, LSD1 represses the transcription of *PGC-1α by* removing the methyl group from mono-methylated and di-methylated lysine 4 of histone H3 (H3K4) [26, 27]. Lack of flavin adenosine dinucleotide, an essential cofactor in fatty acid oxidation (FAO), downregulates LSD1 and promotes PGC-1*α* expression [26]. Interestingly, PGC-1*α* is able to self-regulate its own transcription through YingYang1 (YY1), in a direct interaction that requires the presence of mTOR, thus stimulating mitochondrial activity [28]. Conversely, RIP140, 160MYP, DNMT3B, PARIS, ATL, and p53 are negative transcriptional regulators of PGC-1*α* [29].

The transcriptional activity of PGC-1*α* is also regulated in cells by several post-translational modifications, including phosphorylation, acetylation, and ubiquitination [15]. These post-translational modifications positively or negatively modulate the stability of PGC-1*α* and affect its ability to recruit other transcriptional coactivators [8]. p38 MAPK, AMP-activated protein kinase (AMPK), Akt, and glycogen synthase kinase 3*β* (GSK3*β*) are the best characterized kinases that regulate PGC-1*α* by phosphorylation. p38 MAPK phosphorylates PGC-1*α* at Thr262, Ser265, and Thr298 [30, 31]. These post-translational modifications promote PGC-1*α* stabilization [31] and enhance PGC-1*α* transcriptional activity by displacing transcriptional suppressor p160 Myb-binding protein [32]. AMPK binds to and activates PGC-1*α* by direct phosphorylation on Thr117 and Ser538. Nevertheless unlike p38 MAPK and AMPK, the kinase activity of Akt and GSK3*β* is associated with the inhibition of PGC-1*α* [33–36]. Akt abrogates PGC-1*α* by either direct phosphorylation at Ser570 [31] or inducing CDC like kinase 2 (Clk2), which, in turn, phosphorylates and downregulates PGC-1*α* activity [37]. GSK3*β* phosphorylates and inhibits PGC-1*α* by promoting its proteasomal degradation [35]. The proteasomal degradation of PGC-1*α* can also be regulated by ubiquitination through S-phase kinase-associated protein 1 (Skp1)/Cullin/F-box-cell division control 4 (SCF^Cdc4^) [38].

In different tissues, PGC-1*α* activity is regulated by acetylation through a key mechanism that acts as a sensor of energy status in cells. Sirtuin 1 (SIRT1) is a histone deacetylase located in the nucleus that responds to change in NAD^+^/NADH ratio [39]. In situations of low energy status, the increase in NAD^+^/NADH triggers the activation of SIRT1 and activates PGC-1*α* through its deacetylation [40, 41]. However, when energy abounds in cells, histone acetyltransferase GCN5 catalyses acetylation and promotes PGC-1*α* inhibition [42, 43]. This mechanism, which is addressed in more detail in the following sections, directly associates PGC-1*α* with metabolic regulation.

### 2.3. Tissue-Specific Metabolism Action of PGC-1*α*

**Figure 2:**
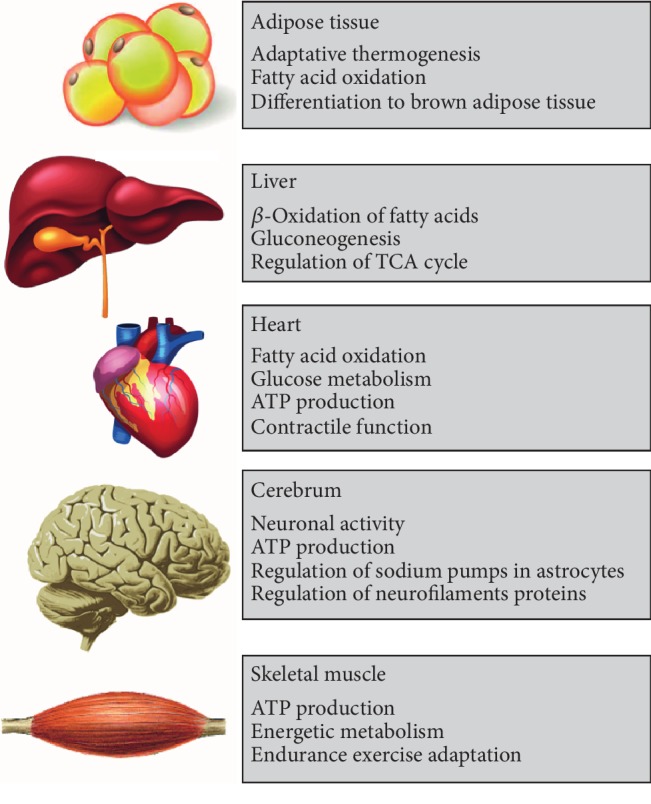
PGC-1*α* metabolic functions.

The main role of PGC-1*α* on the regulation of cellular energy metabolism causes a basal expression in many of body tissues. However, as mentioned above, PGC-1*α* expression is notably enhanced in tissues with high energy demands; e.g., liver, cardiac and skeletal muscle, kidney, brown adipose tissue, brain, or retina (Figure 2) [44]. In these organs, PGC-1*α* activation is specifically regulated by different stimuli, such as physical activity in cardiac and skeletal muscle, cold exposure in brown adipose tissue, and fasting in the liver [11]. Once activated, PGC-1*α* induces transcriptional networks that control the mitochondrial biogenesis and oxidative phosphorylation of energy substrates, which results in tissue-specific gene programs that adjust endurance exercise adaptation in skeletal muscle, thermogenesis in brown adipose tissue, or lipid metabolism and hepatic gluconeogenesis [45, 46].

#### 2.3.1. Adipose Tissue

PGC-1*α* is a molecular switch that induces key adaptive thermogenic program components in brown fat, which involves the activation of mitochondrial fatty-acid oxidation, fuel intake, and heat production by uncoupling protein-1 (UCP1) expression [1]. PGC-1*α* expression is necessary to promote differentiation to the brown-fat lineage, as shown by the induction of UCP1 expression [1]. Remarkably, mice deficient in PGC-1*α* are cold-sensitive due to inefficient thermogenesis, probably caused by impaired fatty-acid *β*-oxidation and electron transport, accompanied by diminished UCP1 induction [47]. Notwithstanding, PGC-1*α* is dispensable for brown adipocyte differentiation [11].

#### 2.3.2. Liver

The maintenance of carbohydrate and lipid homeostasis is vital for the survival of mammalians. As a result, glucose and lipid levels are tightly regulated by an adequate response to environmental variables, such as food intake, stress, physical activities, and temperature [16]. As previously mentioned, PGC-1*α* contributes to the regulation of lipid and carbohydrate metabolism through the *β*-oxidation of fatty acids and hepatic gluconeogenesis, respectively [48].

The liver is the major producer of glucose through two different pathways: glycogenolysis and gluconeogenesis. This second pathway, gluconeogenesis, involves the *de novo* synthesis of glucose from precursors, such as alanine, lactate, pyruvate, and glycerol. Gluconeogenesis is hormonally controlled by the expression and activities of three key enzymes: glucose-6-phosphatase (G-6-Pase), phosphoenolpyruvate carboxykinase (PEPCK), and fructose-1,6-bisphosphatase. In particular, gluconeogenesis occurs in fasting or diabetic states because insulin is low or the liver is resistant to insulin. Yoon et al. have reported a very low PGC-1*α* mRNA expression in the liver of mice. Nevertheless, they observed that PGC-1*α* expression in the liver increased dramatically by fasting [16]. In addition, PGC-1*α* has also been induced in the liver of a type 1 diabetes experimental model and in ob/ob mice, a type 2 diabetes model with high insulin levels, but intense insulin resistance [45]. Indeed mice with liver-specific mutations in the insulin receptor present high PGC-1*α* levels in the liver, which demonstrates the key role of insulin as a suppressor of PGC-1*α* [45, 49]. Once PGC-1*α* is induced, it coactivates a variety of transcription factors, such as FoxO1, glucocorticoid receptor, and hepatocyte nuclear factor 4 alpha (HNF4*α*), in the promoter regions of gluconeogenic genes, PEPCK, G-6-Pase, and fructose 1,6-bisphosphatase and, consequently, increases the transcription of these genes [50]. In these animal models, glucagon via cAMP and glucocorticoids are the major positive factors that trigger the genes of gluconeogenesis in the liver. In fact, administering 8-bromo-cAMP in hepatocytes induces the mRNA expression of PEPCK and G-6-Pase. Moreover, dexamethasone enhances PGC-1*α* gene expression only slightly but significantly synergizes with 8-bromo-cAMP during the induction of PGC-1*α* mRNA [45].

Lipid metabolism in the liver includes several pathways that are interdependent and can be summarized by three processes: acquisition of lipids (uptake of lipids and fatty acids and fatty acid synthesis), lipid storage (triglyceride synthesis and formation of lipid droplets), and lipid consumption (lipolysis and *β*-oxidation). The fed-to-fasted transition promotes metabolic changes in the liver that cause adaptation to nutrient deprivation. These metabolic changes consist essentially in the activation of hepatic gluconeogenesis, the *β*-oxidation of fatty acids, and the synthesis and secretion of ketone bodies [51]. *In vivo* and *in vitro* studies in hepatocytes have revealed that PGC-1*α* is necessary for activating hepatic fasting responses, which include fatty acid *β*-oxidation [52]. In the fasted state, SIRT1 deacetylates PGC-1*α*, and its activity increases [53]. Consequently, PGC-1*α* can interact with nuclear receptors PPAR*α*, estrogen-related receptor alpha (ERR*α*), and HNF4*α* to promote mitochondrial FAO [52, 54, 55]. Accordingly, hepatocyte deletion of SIRT1 diminishes the expression of *β*-oxidation genes, increases fasting-induced lipid accumulation in the liver, and triggers diet-induced steatosis [56, 57]. However in the fed state, insulin stimulates PGC-1*α* phosphorylation by Akt, which impairs the capacity of PGC-1*α* to trigger fatty acid *β*-oxidation [33]. Moreover, hepatic lipin1a interacts with PGC-1*α* and positively controls the fasted genes of FAO [58]. Hatazawa *et al.* have recently observed that PGC-1*α* also increases the expression of several tricarboxylic acid cycle genes [59].

#### 2.3.3. Heart

PGC-1*α* is a critical regulator of oxidative metabolism in the heart due to extremely dynamic and high requests for ATP. This supply fundamentally comes from FAO, although glucose can also be a substrate for mitochondrial oxidation [40]. PGC-1*α* deficiency in the heart implies smaller cardiac reserves in response to chemical or electrical stimulation and is, therefore, less able to do work in response to higher demands [60]. In mice with PGC-1*α* deficiency, impaired left ventricular function and abnormal heart rates after exercise have also been observed [47].

#### 2.3.4. Brain

PGC-1*α* deficiency leads to marked hyperactivity related with axonal degeneration in the brain, mainly in the striatum [61]. Large amounts of ATP are consumed by neuron cells to preserve their axonal transport and ionic membrane gradient, which completely depend on oxidative metabolism to obtain energy for this function [62]. Moreover, PGC-1*α* also modulates key pathways for neuronal function. In fact, the expression of the *α*2 subunit of sodium pumps in astrocytes and the levels of neurofilament proteins lower in the PGC-1*α* null brain [61].

### 2.4. PGC-1*α* and Mitochondrial Biogenesis

Mitochondrial mass is modulated by the dynamic equilibrium between degradation and biogenesis. Mitochondrial biogenesis is a complex process, which involve the activation of different signaling pathways that may modify mitochondrial function. Particularly, these pathways act about control of cell metabolism as a physiological response to increased energy demand as well as in regulation of mitochondrial ROS production and detoxification, in the proper mitochondrial respiratory complexes assembly and in the regulation of potential mutations in the mitochondrial DNA [63, 64].

Mitochondrial and nuclear genes are involved in mitochondrial biogenesis, and it is mediated primarily by the activation of PGC-1*α* [65]. PGC-1*α* leads to the activation of several transcription factors including nuclear respiratory factors (NRF-1 and 2), PPARs, mitochondrial transcription factor A (Tfam), and ERR*α* to increase transcription of genes related to mitochondrial biogenesis and function [66, 67].

NRF-1 and NRF-2 lead to the increase in transcription of key mitochondrial enzymes, and they have been shown to interact with Tfam, which triggers transcription and replication of mtDNA [68]. Mitochondrial biogenesis and PGC-1 were also established by observing mtDNA in mouse C2C12 myotubes expressing PGC-1*α*. Indeed, it was observed that PGC-1*α* induces respiration and mitochondrial biogenesis in muscle tissue by an induction of UCP-2 and trough induction of NRF-1 and NRF-2 gene expression. Another experiment established the relationship between mitochondrial biogenesis and exercise by an acute swimming bout, which increased PGC-1*α* protein expression and NRF-2 binding to the cyclooxygenase (COX) IV promoter and NRF-1 binding to the *δ*-ALAS (*δ*-aminolevulinate synthase) promoter [69]. Furthermore, PGC-1*α* binds to and activates the transcriptional activity of NRF-1 on the promoter of Tfam [70]. As mentioned above, PGC-1*α* also interacts with and coactivates other transcription factors such as ERR*α*, ERR*γ*, thyroid hormone, PPARs, and glucocorticoids [71]. ERRs are nuclear receptors that target in several features of energy homoeostasis, including glucose and lipid metabolism, as well as mitochondrial biogenesis [72] .

Mitochondrial function and the synthesis of ATP are indispensable in cells under normal conditions. Inevitably, mitochondrial activity generates toxic products when a small percentage of electrons do not complete the whole series and instead directly leak to oxygen, resulting in the formation ROS [73]. During the OXPHOS process, there are eleven production sites of free radicals; six are in complex I (NADH/NAD+) and five in complex III at the redox potential of the ubiquinol/ubiquinone (QH2/Q) [74]. These mitochondrial ROS may produce cellular dysfunction but can also act as signaling molecules to activate pro-growth responses linked with several essential cellular signaling processes of growth regulation, differentiation, proliferation, and apoptosis [75, 76]. Consequently, the cell's metabolic state strongly impacts the capacity of ROS production by mitochondria in a PGC-1*α*-dependent mechanism [77, 78].

### 2.5. PGC-1*α* and Antioxidant Defense

PGC1*α* regulates the expression of mitochondrial antioxidant defense in cells. PGC-1*α* increases the levels of manganese superoxide dismutase (MnSOD/SOD2), catalase, peroxiredoxin (Prx) 5, Prx3, UCP-2, thioredoxin reductase (TRXR) 2, and thioredoxin (TRX) 2 and consequently protects cells from mitochondrial dysfunction [79, 80]. Indeed, the upregulation of antioxidant defense by PGC1-*α* has been found essential to prevent cell death associated with mitochondrial failure [79]. In fact, in different cancer cells, PGC-1*α* upregulation promotes cell survival by protecting cells from excessive mitochondrial ROS generation [81, 82]. PGC-1*α* is positively upregulated when cells are exposed to oxidative stress in order to prevent oxidative damage [83]. Accordingly, the lack of PGC1*α* is associated with a higher susceptibility to oxidative damage in mice [5, 61, 84]. Interestingly, even PGC-1*α* heterozygote (PGC-1*α* (+/-)) mice failed to augment *Sod2* mRNA and protein levels in liver after peritonitis induction, which led to increased levels of mitochondrial oxidized glutathione (GSSG)/reduced glutathione (GSH) ratio and protein carbonyls in these mice [85]. Importantly, the upregulation of PGC-1*α*-antioxidant target genes was found not only crucial to prevent oxidative damage but also to decrease mitochondrial ROS levels and to ensure mitochondrial integrity during cell differentiation [86].

The upregulation of mitochondrial antioxidant gene program through PGC-1*α* is closely associated with FoxO proteins. FoxO proteins by itself also protect from oxidative stress by binding to the promoters of *SOD2*, *catalase*, and *Prdx3* genes [87]. Interestingly, FoxO3 is a direct target of PGC-1 and physically interacts with PGC-1*α* in vascular endothelial cells to promote mitochondrial oxidative stress protection through a mechanism regulated by the activity of SIRT1 [80, 88]. Inactivation of Foxo3a and subsequent downregulation of PGC-1*α* result in reduced ROS detoxification capacity, which promotes endothelial cell migration [23].

It is noteworthy that the antioxidant function of PGC-1*α* is paired with its role enhancing the mitochondrial electron transport and mitochondrial mass in cells with high energy demands. Therefore, the regulation of mitochondrial antioxidant defense through PGC-1*α* is considered an adaptive mechanism to ensure an adequate response to metabolic requirements avoiding the cytotoxic effects of ROS accumulation [89].

#### 2.5.1. Regulation of PGC-1*α* through ROS/RNS

**Figure 3:**
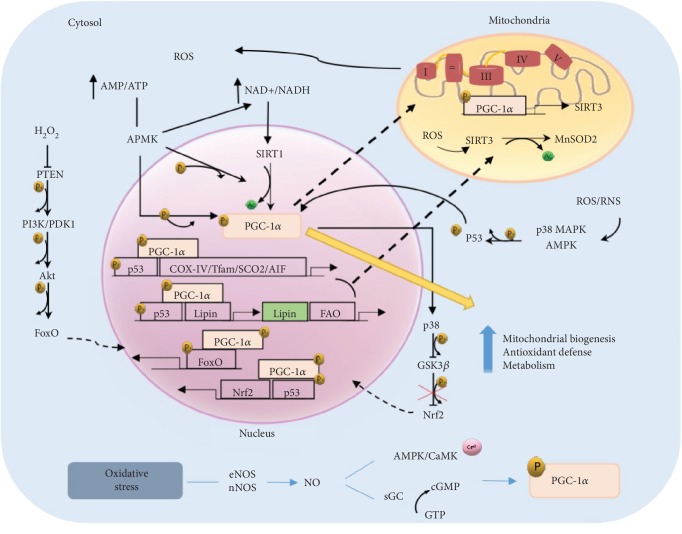
PGC-1*α* signaling pathway in response to ROS/RNS.

In addition to the transcriptional and post-translational regulation of PGC-1*α* expression and activity, both ROS and reactive nitrogen species (RNS) may modulate the different functions of PGC-1*α* (Figure 3). AMPK is an energy sensor that is induced in low-energy situations by detecting decrease in the AMP/ATP ratio. Structurally, AMPK has cysteine residues in the *α*-subunit that respond to mitochondrial ROS and are responsible for its activation [90]. Once activated, AMPK phosphorylates PGC-1*α* leading to increase in glucose transport, fatty acid oxidation, and mitochondrial biogenesis [91, 92].

The NAD^+^/NADH ratio is a sensitive marker of the cellular redox state [93]. In fact, ROS production by activity of complex I depends on the matrix redox potential, and as a consequence, the increase in NAD^+^/NADH triggers PGC-1*α* deacetylation through SIRT1 activation [94]. In addition, AMPK may increase the level of NAD^+^, producing SIRT1 phosphorylation and thereby promoting mitochondrial biogenesis [95]. On the other hand, SIRT3, another member of the SIRT family, is found in the mitochondria. It is a target of PGC-1*α* that is increased in mitochondria by activation of SIRT1 [96]. Strikingly, SIRT3 deacetylates and activates MnSOD during the presence of mitochondrial ROS, and therefore, it is a key player in the antioxidant program [29].

PI3K/Akt pathway is downstream of phosphatase and tensin homolog (PTEN) and is frequently activated under oxidative stress conditions. In turn, H_2_O_2_ may inhibit PTEN phosphatase activity by redox modulation [97, 98]. Thus, PTEN acts phosphorylating PI3K and thereby allows Akt phosphorilation. Hence, high levels of H_2_O_2_ promote Akt phosphorylation due to PTEN redox-mediated inactivation. Once activated, Akt may directly phosphorylate FoxO family, which participate in cellular transcription programs including metabolism, ROS detoxification, and mitochondrial biogenesis trough PGC-1*α* [29, 99].

The complex role of PGC-1*α* and its ability to strike a correct balance between energy requirements and ROS protection may be understood by considering the relation with antioxidant transcription factor nuclear factor erythroid 2-related factor 2 (Nrf2). PGC-1*α* activates Nrf2 via inhibition of GSK3*β*. It is known that GSK3*β* avoids the translocation of Nrf2 to the nucleus by phosphorylation. In oxidative stress conditions, GSK3*β* is inactivated by p38, which is positively regulated by PGC-1*α*, and as a consequence, the antioxidant defense is activated by Nrf2 [100, 101].

As we commented above, PGC-1*α* regulation may be due to RNS [102]. Nitric oxide (NO) is synthesized by three isoforms of NO synthase (NOS) that depend of tissue localization. These isoforms utilize l-arginine and oxygen as substrates in almost all mammalian cells [103]. Neuronal NOS (nNOS) is constitutively expressed in neuronal tissue, and the endothelial NOS (eNOS) is mostly expressed in endothelial cells. Both are constantly active in response to stimuli, such as oxidative stress, that induce an increase in the intracellular Ca^2+^ concentration [104]. Inducible NOS (iNOS) is found in macrophages, neutrophils, microgial, and astroglial cells and is expressed in response to different factors such as cytokines and bacterial lipopolysaccharide [105]. In normal conditions, NO levels produced by eNOS and nNOS are low [106, 107]. However, studies with mice deficient in eNOS and nNOS showed mitochondrial mass markers and decreased PGC-1*α* levels [20, 108].

NO stimulates mitochondrial biogenesis through generation of cyclic guanosine monophosphate (cGMP) by soluble guanylate cyclase (sGC) that activates PGC-1*α* [95]. Depending on tissue localization, NO regulation is different. For instance, in brown adipose or muscle tissue, several studies with eNOS knockout mice show that NO/cGMP pathway controls mitochondrial biogenesis [109, 110]. However, in endothelial cells, NO is necessary to migration cells [111]. NO/cGMP produces PGC-1*α* downregulation through FoxO3 inactivation in a mechanism that requires induction of PI3K/Akt [112]. Its inactivation leads to transcriptional downregulation of PGC-1*α* and, as a consequence, a reduced ROS detoxification and induction of endothelial migration [23]. Another important independent regulator of sGC by NO is AMPK [113]. AMPK in situations with low energetic levels as hypoxia, exercise, or increase ROS and NO is activated and produces upregulation of PGC-1*α* that leads to an antioxidant response and improves metabolic state [46, 114, 115]. In addition, CaMK protein is regulated by NO. It is a calcium-dependent kinase that resides near intracellular compartments with high levels of calcium. In high contractile activity of skeletal muscle, NO levels increase and induce mitochondrial biogenesis by CaMK due to high cytosolic calcium concentration. These events lead to activation of various transcription factors such as NRF-1 and 2 and PPAR-*α*, which produces an increase in the expression of proteins of the respiratory chain, upregulates the levels of enzymes of beta oxidation, and activates expression of the mitochondrial genome [116].

On the other hand, a relevant protein that is activated in oxidative stress is p53, which depending on the levels of ROS/RNS may act as metabolic regulator or even inducing apoptosis [83, 117]. In acute physical exercise, increase of ROS produces transcriptional signaling that leads to mitochondrial biogenesis and improves the oxidative capacity of skeletal muscle through p53. For instance, AMPK and p38 MAPK may phosphorylate p53 and induce its binding to response elements within the PGC-1*α* promoter that lead to the expression of nuclear genes encoding mitochondrial proteins such as Tfam, COX-IV, SCO2, and AIF [118]. In situations of caloric restriction, p53 colocalizes in nucleus with PGC-1*α* to enhance Lipin-1 expression, regulator of fatty acid metabolism, to increase fatty acid oxidation and triglyceride synthesis. Moreover, phosphorylated p53 may interact with PGC-1*α* to enhance Nrf2 and MnSOD antioxidant expression and reduce the effects of ROS/RNS [119, 120]. Antioxidant defense may also be activated by low levels of GSH or increased NO/cGMP, which leads to the activation of p53 [121, 122]. On the other hand, p53 may be repressed transcriptionally by GLUT-1 and GLUT-4 genes in situations such as excess caloric intake that cause reduction of PGC-1*α*, NRF-1, and Tfam activation [123, 124]. Definitely, p53 leads to an upregulation of antioxidant production, cellular senescence, regulate fatty acid oxidation, and oxidative phosphorylation energy pathways, while reducing glycolytic and apoptotic signaling by PGC-1*α* [83].

To conclude, mitochondrial ROS/RNS production is modulated by many factors including mitochondrial electron transport chain efficiency, mitochondrial antioxidant content, disponibility of oxygen, NO concentrations, availability of metabolic electron donors, UCP activity, cytokines, and vasoactive agonists affecting several disease such as diabetes, cardiovascular diseases, ischemic stroke … in which PGC-1*α* is a crucial factor to improve the metabolic states reducing oxidative stress effect in these pathologies [125, 126].

## 3. PGC-1*α*, Inflammation, and Oxidative Stress

PGC-1*α* activity is closely related to inflammatory processes. Under inflammatory conditions, PGC-1*α* levels are downregulated, which decisively contributes to enhance the inflammatory response [4, 6, 127]. Recently, we found that severity of acute pancreatitis in obese mice is associated with PGC-1*α* levels. Thus, mice lacking PGC-1*α* exhibited increased levels of inflammatory infiltrate in the pancreas with pancreatitis [5]. It has been previously reported that the severity of sepsis-associated acute kidney injury (AKI) correlates with PGC-1*α* levels in the kidney, and accordingly, lack of PGC-1*α* causes persistent kidney injury following endotoxemia [128]. In fact under the basal condition, PGC-1*α* KO mice display spontaneous subclinical kidney injury characterized by tubulointerstitial inflammation and exhibit a higher mortality rate than their wild-type (WT) littermates after AKI induction [129]. In addition, the expression of PGC-1*α* diminishes in aging muscle and has been associated with systemic inflammation in mice [130].

Several studies have reported the key role of cytokines in regulating PGC-1*α* levels under inflammatory conditions. In tubular cells, tumor necrosis factor-*α* (TNF-*α*) reduces the expression of PGC-1*α*, and in a similar fashion, PGC-1*α* is downregulated in C2C12 muscle cells upon TNF-*α* and interleukin 1*β* (IL1-*β*) treatment [128, 131]. Similar findings have been reported in cardiac cells, where both lipopolysaccharide (LPS) and TNF-*α* reduce PGC-1*α* levels [132, 133]. LPS is a well-documented factor that dramatically lowers PGC-1*α* levels in different tissues, such as heart, kidney, muscle, and liver [134]. Nevertheless, it is noteworthy that the regulation of PGC-1*α* levels by LPS seems tissue specific. In skeletal muscle, LPS induces PGC-1*α* expression upon short-term exposure but lowers its transcript levels 24 h after LPS injection [135]. In contrast, LPS early lowers PGC-1*α* in the liver, but its expression recovers 8–16 h after injection [135].

Although the molecular mechanisms driving PGC-1*α* downregulation during inflammation should be clarified, the activation of the nuclear factor kappa-light-chain-enhancer of activated B cells (NF-*κ*B) signaling pathway seems to play a critical role. Indeed, the repression of PGC-1*α* expression under inflammatory conditions is rescued by NF-*κ*B inhibition [133, 136, 137]. TNF-*α* represses PGC-1*α* gene expression by activating both NF-*κ*B and p38 MAPK [133]. The activation of interleukin 1 receptor associated kinase 1 (IRAK-1), an upstream kinase on the NF-*κ*B signaling pathway activated through Toll-like receptor/interleukin-1 receptor (TLR/IL-1R), lowers PGC-1*α* levels in LPS-stimulated macrophages [138].

PGC-1*α* improves the balance between ROS production and its detoxification during inflammation by regulating key antioxidant gene expression [139]. Therefore, low PGC-1*α* levels in inflamed tissues increase ROS production and cause oxidative damage [5, 86, 140, 141]. PGC-1*α* also participates in the regulation of macrophage polarization from the proinflammatory M1 to the anti-inflammatory M2 type. Thus, altered PGC-1*α* levels could promote a skewed balance of macrophages during inflammation [127]. After LPS/TNF-*α* injection, the overexpression of PGC-1*α* in skeletal muscle does not suppress proinflammatory cytokine expression but lowers M1 cytokine IL-12 levels and enhances the production of anti-inflammatory M2 cytokines [142]. In addition, PGC-1*α* KO mice exhibit increased M1 and decreased M2 responses in the kidney after AKI induction [129]. Although the molecular mechanisms regulating macrophages polarization are poorly understood, these cells have been reported to be metabolically reprogrammed during polarization [143]. So the PGC-1*α*-dependent regulation of mitochondrial function would play an essential role here [144]. Intriguing accumulated evidence suggests a critical role of ROS in regulating macrophage polarization [145], but the specific contribution of ROS levels controlled by PGC-1*α* during this process is an exciting question yet to be addressed.

Together with the antioxidant capacity and the macrophages phenotype, PGC-1*α* also regulates the levels of proinflammatory cytokines during inflammation. In human aortic smooth muscle and endothelial cells, PGC-1*α* decreases TNF-*α*-induced vascular cell adhesion molecule 1 (VCAM-1) and monocyte chemoattractant protein 1 (MCP-1) expression [146]. In skeletal muscle, lack of PGC-1*α* induces the expression of TNF-*α*, IL-6, and CD68 [147], and accordingly, PGC-1*α* diminishes the increase in proinflammatory cytokine expression elicited by TNF-*α*, TLR agonists, and saturated free fatty acids in C2C12 muscle cells [3]. In fact, *chronic obstructive pulmonary disease (COPD)* patients exhibit high TNF-*α* levels in skeletal muscle, which correlates with lower PGC-1*α* levels [148]. Moreover, TNF-*α* levels rise in the skeletal muscle of mice exposed to cigarette smoke, while PGC-1*α* levels lower [149].

Mechanistically speaking, levels of proinflammatory cytokines are regulated by PGC-1*α* through its physical interaction with the p65 NF-*κ*B subunit. Consequently, PGC-1*α* blocks NF-*κ*B transcriptional activity toward its target genes, including those encoding proinflammatory cytokines [5, 150, 151]. In human cardiac cells and mouse heart, p65 has been found to be constitutively bound to PGC-1*α*. Remarkably, this binding is enhanced upon TNF-dependent NF-*κ*B activation [150]. In acute pancreatitis, we found that PGC-1*α* binds p65 and phospho-p65 in the pancreas, and the complex with the latter is more abundant during pancreatitis [5]. Lack of PGC-1*α* during pancreatitis specifically upregulates IL-6 [5], a reliable marker of severity in acute pancreatitis [152, 153]. Hence, we proposed that PGC-1*α* selectively modulates NF-*κ*B transcriptional activity by acting as a specific NF-*κ*B repressor toward IL-6 during acute pancreatitis [5]. Furthermore, NF-*κ*B is a redox sensitive factor, and thus, the PGC-1*α*-dependent regulation of antioxidant genes can modulate NF-*κ*B activation during inflammation [139, 154]. In astrocytes, enhanced PGC-1*α* levels augment mitochondrial antioxidant capacity and decrease the production and secretion of both IL-6 and chemokine (C-C motif) ligand 2 [155]. Silencing PGC-1*α* in skeletal muscle drastically downregulates superoxide dismutase 1 (SOD1) expression, increases oxidative stress, causes energy deficiency, augments the serum levels of proinflammatory cytokines, and induces muscle fibrosis, the major pathological feature of amyotrophic lateral sclerosis [151]. These changes have also been observed in *SOD1* (G93A) transgenic mice, which exhibit low PGC-1*α* levels [151].

**Figure 4:**
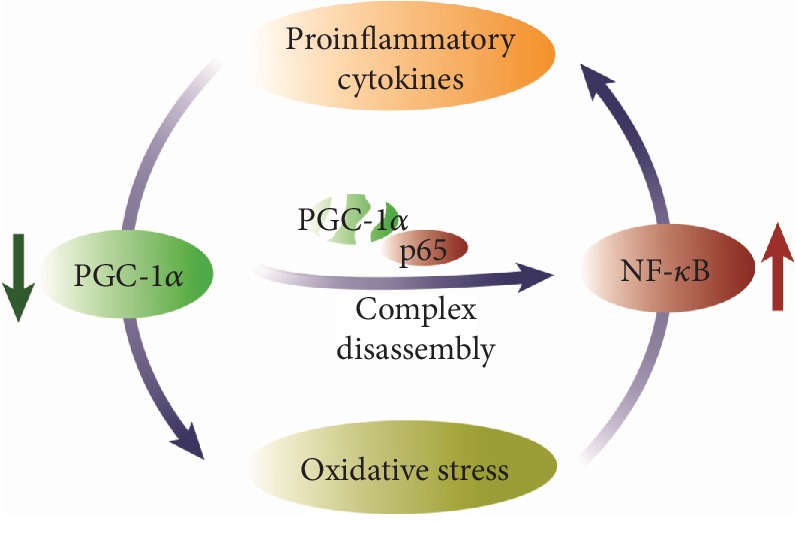
Redox regulation of p65 activity by PGC-1*α* interaction.

Taken together, PGC-1*α* and NF-*κ*B are mutually regulated during inflammation and in a vicious cycle where oxidative stress plays an essential role (Figure 4). NF-*κ*B activation lowers the expression of PGC-1*α* during inflammation, and low PGC-1*α* levels downregulate its antioxidant target genes by promoting oxidative stress. Reciprocally, low levels of PGC-1*α* and concomitant oxidative stress promote NF-*κ*B activation by thereby exacerbating inflammatory response.

## 4. PGC-1*α* and Metabolic Syndrome

In recent decades, society is characterized by a sedentary lifestyle and changes in food habits, which means that the prevalence of being overweight and obese constantly increases. In addition, obesity is associated with type 2 diabetes mellitus (T2DM), cardiovascular disease, and fatty liver disease. These pathological conditions are clustered in a syndrome called “metabolic syndrome” and are associated with abdominal obesity, high blood sugar levels, increased blood pressure, and abnormal triglyceride and cholesterol levels [156]. In these conditions, a state of chronic low-grade inflammation is commonplace, along with altered redox control, which may contribute to the etiology and development of these metabolic abnormalities [157].

As previously mentioned, PGC-1*α* is a powerful regulator of cellular and systemic metabolism and is responsible for the organism's nutritional state. In this context, dysregulation of PGC-1*α* activities may modify the metabolic properties of tissues and might, therefore, be involved in different metabolic disorders (Table 1). In fact, single nucleotide polymorphisms in the human PGC-1*α* gene have been shown to be associated with obesity, diabetes, and hypertension [158].

### 4.1. PGC-1*α* and Mitochondrial Dysfunction in Obesity

Obesity consists of excessive fat accumulation in adipose tissue as a result of a chronic positive energy balance [159]. Adipose tissue dysfunction in obesity is closely related to inflammation owing to diminished mitochondrial capacity in adipocytes [160]. In recent years, impaired mitochondrial oxidative metabolism has been considered a molecular hallmark of obese adipose tissue [161]. Mitochondrial biogenesis and mitochondria-related transcription reduce in both obesity and diabetes experimental models [162]. In genetic mouse or diet-induced obesity models, limited OXPHOS capacity has been observed in white adipocytes in both the presence and absence of glucose intolerance. To conclude, mitochondrial dysfunction is associated with obesity but not with glucose tolerance [163]. Interestingly, mitochondrial dysfunction may potentially exacerbate ROS production and can also diminish the availability of ATP, which is indispensable for the transcription of antioxidant and DNA-repair genes and, therefore, leads to oxidative stress [164]. Moreover, the genes encoding mitochondrial proteins reduce in obese mice without diabetes, which are upregulated after rosiglitazone treatment [165]. However, another study found low levels of mitochondrial DNA, OXPHOS mitochondrial protein subunits, and a number of mitochondria in diabetic, but not obese, mice [166].

Similarly, several human studies have linked mitochondrial dysfunction with both obesity and PGC-1*α* [167–169]. In another study, the gene expression of PGC-1*α* and the OXPHOS protein subunit levels was downregulated in isolated primary mature adipocytes of co-twins with obesity in relation to their lean co-twins when distinguishing the characteristics of obesity acquired from possible genetic effects [170]. Furthermore, dramatic upregulated inflammatory gene expression has been found in adipocytes and the adipose tissue of obese co-twins. This finding may suggest that the suppression of mitochondrial oxidative phosphorylation and the activation of inflammatory pathways may be biologically linked [170]. One striking finding was that hypermethylation has been observed in the body of the PGC-1*α* gene at two CpG sites in obese co-twins, which correlates with low PGC-1*α* expression [161].

Recent evidences support the importance of mitochondrial dynamics in mitochondrial function and cellular metabolism [171, 172]. Indeed, the balance in fusion/fission processes is directly associated with the regulation of the bioenergetic state of the cell. Mitochondrial fusion is related with optimized mitochondria, while mitochondrial fission is associated with impaired mitochondria and their elimination by auto- or mitotophagy [173]. The main proteins involved in fusion process are autosomal dominant optic atrophy-1 (OPA1) and mitofusins 1 and 2 (Mfn1 and Mfn2). In contrast, the major proteins involved in mitochondrial fission are dynamin-related protein 1 (DRP1) and fission protein 1 (FIS1) [174].

As mentioned above, mitochondrial dynamics may act as a crucial regulator in the pathophysiology of obesity-related diseases. Indeed, reduced Mfn2 expression was described in obese type 2 diabetic patients, leading to functional and morphological fragmentation of the mitochondrial network [175]. Obese Zucker rats also showed a reduction in Mfn2 expression and mitochondrial size accompanied by insulin resistance and a reduced glucose uptake [176]. On the other hand, Drp1 and Fis1 were increased in liver from db/db mouse as well as in mice with high-fat diet-induced obesity, resulting in impaired mitochondrial function [177]. Furthermore, in skeletal muscle of mice with genetic obesity and with diet-induced obesity, it was also observed mitochondrial fragmentation and increased mitochondrion-associated Drp1 and Fis1 [177]. Strikingly, PGC-1*α* induces the transcriptional activity of the human Mfn2 gene promoter [178]. In fact, PGC-1*α* overexpression enhances Mfn2 expression in cultured muscle cells with other genes typically induced by PGC-1*α*, such as *β*-ATP synthase, COX-II, and cytochrome c [178]. Holmström and coauthors observed decreased protein PGC-1*α* expression and diminished mitochondrial respiratory capacity in the liver of obese diabetic (db/db) mice, which coincides with decreased mitochondrial fusion and increased mitochondrial fission [179]. In conclusion, these reports suggest that an imbalance between fusion and fission processes is related to the characteristic mitochondrial dysfunction of obesity-related metabolic diseases and that PGC-1*α* plays a key role in these processes.

### 4.2. Type 2 Diabetes Mellitus

The global rise in obesity is accompanied by a comparable increase in the incidence of T2DM, which is estimated to affect some 415 million people today [180]. T2DM is characterized by insulin resistance in target organs and relative insulin deficiency due to pancreatic *β*-cell dysfunction. Recent evidence indicates a marked association between insulin resistance and mitochondrial dysfunction accompanied by a significant drop in the mRNA levels of PGC-1*α* [181]. Nevertheless, increased PGC-1*α* activity contributes to an increase in hepatic glucose output and to hyperglycemia progressing [182]. Indeed the knockdown of PGC-1*α* in the liver improves glucose tolerance and hepatic insulin sensitivity in db/db mice [183]. Hence, modulating PGC-1*α* expression and activity may have important implications for systemic glucose homeostasis.

Several studies have emphasized high ROS levels in diabetic patients and the role of ROS in cellular signaling alteration, which contributes to the development and complications of T2DM [184]. As previously mentioned, mitochondrial dysfunction is associated with T2DM, and this mitochondrial impairment may trigger oxidative stress [164]. Therefore, PGC-1*α* and oxidative stress are involved in T2DM.

Vanin-1 (VNN1) is a hepatic oxidative stress sensor whose levels increase in the blood and urine of diabetic patients [185]. VNN1 may overactivate gluconeogenesis under the control of the PGC-1*α*/HNF-4*α* complex by contributing to uncontrolled hyperglycemia [186]. Furthermore, amino-thiol cysteamine generation due pantetheinase VNN-1 activity is associated with reduced *γ*-glutamylcysteine synthetase activity in the liver, which leads to less stored GSH [187]. In hepatocytes, increased asymmetric dimethylarginine (ADMA), an endogenous inhibitor of NOS, contributes to hepatic mitochondrial dysfunction in streptozotocin-induced diabetic rats, accompanied by inhibitions of PGC-1*α* transcription, mitochondrial biogenesis, and NO synthesis and also by rising MDA levels [188]. Interestingly, these ADMA effects may be inhibited by treatments with an antioxidant like pyrrolidine dithiocarbamate [188]. In line with this, another report shows that the downregulation of GLUT2 and PGC-1*α* triggered by glucose oxidase is evidently inhibited by NAC in hepatocytes of rats [189]. The expression of Sirt3 and its associated transcription factor, FOXO3a, in adipocytes and skeletal muscle dramatically decreases in experimental models of diabetes [190]. These low expressions induce oxidative stress due to a reduced binding affinity of FoxOA3 to the PGC-1*α* promoter, which may subsequently cause mitochondrial membrane damage and lead to insulin resistance [190]. SIRT3 overexpression might stimulate FOXO3a deacetylation and, consequently, PGC-1*α* and MnSOD upregulation, which enables ROS detoxification and improves insulin sensitivity [190].

Oxidative stress plays a key role in the development of diabetes complications. High glucose treatment in rat glomerular mesangial cells results in the downregulation of PGC-1*α*, increased mitochondrial ROS generation, and mesangial cell hypertrophy. These pathological changes are reversed by the transfection of pcDNA3-PGC-1*α*, which suggests the protective role of PGC-1*α* in diabetic nephropathy [191]. Choi et al. report that PGC-1*α* overexpression may prevent high glucose-induced oxidative stress in dorsal root ganglion neurons [192]. In *ob/ob* cardiomyocytes, mitochondrial biogenesis is impaired, as demonstrated by low Ppargc-1a/Nrf-1/Tfam mRNA levels and AMPK phosphorylation coupled with increased PGC-1*α* acetylation [193]. In fact, sulforaphane administration may prevent T2DM-induced cardiomyopathy by reversing the oxidative stress-induced inhibition of the LKB1/AMPK/PGC-1*α* signaling pathway [194]. Accordingly, caloric restriction presented cardioprotective effects through SIRT1/PGC-1*α* activation in *ob/ob* mice with cardiac hypertrophy [195]. Finally, a recent study into human retinal microvascular endothelial cells has demonstrated that high glucose stimulation increases miR-34a expression, which induces cellular senescence by mitochondrial dysfunction and loss of antioxidant capacity through PGC-1*α* deficiency [196].

Taken together, these results indicate a significant cooperative role of PGC-1*α* and oxidative stress in the pathogenesis of diabetes.

### 4.3. Hepatic Steatosis

Obesity is associated with fatty liver disease or steatosis, which may lead to steatohepatitis. Nonalcoholic fatty liver disease (NAFLD) is defined as excessive amounts of fat accumulating in hepatocytes, which can develop to nonalcoholic steatohepatitis and lead to steatosis, liver inflammation, and liver fibrosis [197]. This pathological condition may progress into liver failure, liver cirrhosis, and eventually to hepatocellular carcinoma [198].

NAFLD is characterized by the impairment of hepatic lipid metabolism, insulin resistance, and redox imbalance [199]. Insulin resistance is closely associated with excessive lipid accumulation, and it is widely accepted that NAFLD is a hepatic manifestation of a systemic impairment of insulin resistance [200]. The cellular redox state may regulate the activity of the enzymes involved in lipid metabolism by generating post-translational modifications directly or by modulating of second messengers or inducing conformational changes to nuclear receptors [201]. Indeed reduced redox status established by redox pairs, such as NADPH/NADP+, NADH/NAD+, and GSH/GSSG [125], may partially suppress *β*-oxidation in liver mitochondria [202].

There is evidence to suggest that mitochondrial dysfunction and decreased fatty acid oxidation play a central role in the pathogenesis of NAFLD [202]. PGC-1*α* expression is lower in the liver of obese humans than in lean people [58]. In fact, *PPARGC1A* methylation status is associated with NAFLD [203]. Accordingly, chronic high-fat diet lowers hepatic PGC-1*α* levels in sedentary WT mice, which leads to reduced hepatic mitochondrial respiration [204]. It is noteworthy that mice with liver-specific PGC-1*α* deficiency exhibit steatohepatitis due to impaired mitochondrial oxidative capacity [205]. PGC-1*α* overexpression increases FAO by high mitochondrial content and reduces triglyceride storage both *in vitro* and *in vivo* [205].

PGC-1*α* is also able to regulate the mitochondrial antioxidant system in the liver [84]. Several studies in humans and experimental models indicate that antioxidant defense mechanisms are disrupted in NAFLD [199]. Reduction in antioxidant factors, including SOD1, catalase, and glutathione, and glutathione S-transferase activities, correlates with liver disease severity [206]. Furthermore, a marker of lipid peroxidation, e.g., 3-nitrotyrosine, has been found in not only NAFLD patients but also in animal models [207, 208]. Peroxidation of mitochondrial membrane components may impair the activity of ETC, ATP depletion, and ROS overproduction [209]. In addition to oxidative damage and ATP depletion, NAFLD development is associated with the induction of cytokines in Kupffer cells and the activation of hepatic stellate cells by contributing to the inflammatory process and favoring the transition of nonalcoholic steatohepatitis to liver fibrosis [141]. These studies indicate a close association between lipid metabolism and antioxidant signaling. Nonetheless, several aspects of these pathways must be further investigated.

### 4.4. Cardiovascular Diseases

Metabolic syndrome is associated with an increased risk of cardiovascular diseases (CVDs) [156]. Actually, CVDs are a leading cause of mortality worldwide [210] and group several conditions of blood vessels and the heart that may lead to peripheral vascular diseases, myocardial infarction, or stroke [200]. Of the different molecular mechanisms involved in these diseases developing, mitochondrial dysfunction accompanied by bioenergetics alterations and oxidative stress is a hallmark of CVDs [210].

PGC-1*α* is expressed in both cardiomyocytes and endothelial cells. Mice genetically deficient in PGC-1*α* have irregular cardiac energetic reserves [60], respond inefficiently to stressful stimuli, such as transverse aortic banding [211], and present poor contractility responses and heart rates [47]. Indeed the cardiomyocytes isolated from PGC-1*α*^−/−^ mice display reduced oxidation of fatty acids and ineffective ATP production [212]. Consistent with this, PGC-1*α*^−/−^ hearts are unable to increase work output in response to inotropic stimulation [212]. Increasing attention has been paid in recent years to the role that PGC-1*α* plays in the vasculature. PGC-1*α* modulates mitochondrial biogenesis and several anti-ROS genes, including catalase, MnSOD, and heme oxygenase [80]. Moreover, PGC-1*α* prevents oxidative injury through AMPK activation and, as a result, protects against endothelial dysfunction [213]. This interesting study suggests that endothelial PGC-1*α* may inhibit endothelial dysfunction to prevent angiotensin II-mediated JNK activation, which is an early feature of atherosclerosis in diabetes, a process that depends on vascular ROS production [213]. PGC-1*α* overexpression in HUVEC cells produces less intracellular ROS and exhibits the inhibition of caspase-3 activation with improved cell viability after oxidative stress induced by H2O2, DMNQ, and high glucose conditions [79]. Interestingly, the suppression of ROS detoxification through the post-translational inhibition of PGC-1*α* with serine 570 phosphorylation may develop vascular hypertrophy induced by angiotensin II [214]. Finally, PGC-1*α* may also prevent ROS production and the apoptosis of endothelial cells by increasing FAO and enhancing the ATP/ADP activity of the adenine nucleotide translocator [215]. These studies collectively indicate that PGC-1*α* plays important roles in the biology of various CDV cell types in both cardiac myofibers and the vascular wall. Research into this topic remains insufficient, and there is still much to learn.

Recent evidence suggests that mitochondrial biogenesis and the ROS-detoxifying system may play a crucial role as endogenous protective mechanisms in cerebral ischemia [216, 217]. In fact, in one interesting experiment, rats trained for 5 days after cerebral ischemia exhibited an increased expression of the genes encoding PGC-1*α* and NRF-1 and presented smaller infarct volumes and diminished neurological deficits compared to sedentary rats [218]. PGC-1*α* gene expression is also induced under ischemia-hypoxia conditions in different tissues [6]. In neuronal cells, PGC-1*α* is required to induce several ROS-detoxifying proteins, including catalase, UCP2, glutathione peroxidase, and MnSOD [89]. The downregulation of PGC-1*α* expression in mice aggravates the harmful effects of either kainic acid on the hippocampus or 1-methyl-4-phenyl-1,2,3,6-tetrahydropyridine (MPTP) on substantia nigra [89]. In addition, pretreatment with PGC-1*α* antisense oligodeoxynucleotide reduces the expression of MnSOD and UCP-2, which leads to exacerbated oxidative injury and increased delayed neuronal death in the hippocampus after transient global ischemia [188]. Instead, enhanced PGC-1*α* expression may protect neural cells from oxidative stress-mediated cell death [89]. The upregulation of UCP-2 after stroke diminishes the release of ROS and decreases neuronal loss in brain tissue, which suggest novel neuroprotection against ischemic brain injury [219]. Accordingly in animal models of transient focal cerebral ischemia, MnSOD overexpression has a protective effect against oxidative stress-induced neuronal damage [220]. Chen et al. have demonstrated that ROS overproduction in hippocampal CA1 neurons may induce the activation of MnSOD and UCP-2 through the PGC-1*α* signaling pathway by providing a neuroprotective effect [221]. It has been recently described how adiponectin upregulates PGC-1*α* levels through the AMPK pathway and improves the excytotoxicity induced by excessive N-methyl-D-aspartate (NMDA) receptor activation [222]. In view of the critical relation between ROS overproduction and ischemia-induced neuronal damage, it is reasonable to argue the importance of PGC-1*α* in this context.

**Figure 5:**
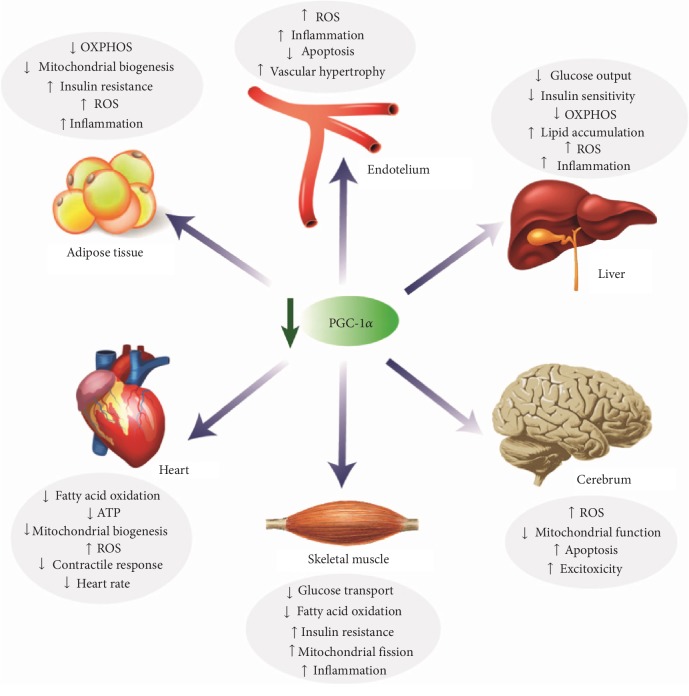
PGC-1*α* downregulation in metabolic syndrome.

In summary, PGC-1*α* is well-known as crucially contributing to the development of metabolic syndrome. PGC-1*α* downregulation may trigger an inflammatory process linked to an altered redox control that can contribute to the development of metabolic disorders in different tissues (Figure 5).

## 5. Metabolic Therapy of PGC-1*α*

PGC-1*α* is profoundly implicated in mitochondrial function, oxidative metabolism, and ROS detoxification, and deregulation of its expression results in metabolic disturbances that may lead to inflammation and metabolic disease. Targeting PGC-1*α* can represent an interesting strategy in metabolic disease, as it is able to modulate metabolic pathways.

Nowadays, numerous drugs have been used to alter PGC-1*α* activity [223]. For instance, in hepatic cells, metformin and 5-aminoimidazole-4-carboxamide ribonucleotide (AICAR) downregulate gluconeogenesis in an AMPK-dependent mechanism. AMPK may inactivate PGC-1*α* through phosphorylation and also allows dissociation of CREB/CBP/TORC2 complex, which, as a consequence, decreases PGC-1*α* transcription, blocks expression of PEPCK and G-6-Pase, and improves sensitivity of liver insulin [224–226]. Furthermore, AMPK may decrease glucose synthesis through FOXO1-mediated repression of PGC-1*α* [227]. However, conclusions from clinical studies prevent talking about a beneficial effect of metformin on liver diseases [228]; surely due to the low regulation of PGC-1*α* that may reduce mitochondrial function and oxidative and proinflammatory environment that further exacerbates hepatic steatosis.

Resveratrol, a polyphenolic compound, may induce both SIRT1 and AMPK, stimulating mitochondrial functions and FAO by activation PGC-1*α* [229]. Interestingly, while low levels of resveratrol are needed to induce SIRT1, higher doses are necessary to activate AMPK [230]. Indeed, treatment with resveratrol of SIRT1 KO mice does not show beneficial properties [230]. Although the effects of resveratrol on hepatic steatosis are questionable, clinical studies reveal that it is beneficial for patients with diabetes, CVDs, neurological disorders, and even in some types of cancer [231].

Epoxyeicosatrienoic acids (EETs) are signaling products formed from arachidonic acid by cytochrome P450 epoxygenases [232]. EETs are important cardiovascular modulators associated with vasodilatory, antiapoptotic, and anti-inflammatory effects [232]. Recently, Singh et al. demonstrate that EET agonist improves cardiomyopathy in an obese mice model of metabolic syndrome through PGC-1*α* upregulation [233]. This study identifies for the first time the use of EET-agonist-mediated induction of PGC-1*α* as a novel pharmacologic target to treat myocardial dysfunction caused by obesity and diabetes mellitus.

PGC-1*α* functions may also be negatively modulated by forskolin, a flavonoid that via SIRT6-mediated GCN5 activation [234]. As we have noted above, GCN5 is an acetyltransferase triggered by caloric excess, which actions with SRC-3 to acetylate PGC-1*α* and decrease expression of genes implicated in gluconeogenesis and FAO [43]. Indeed, mice with SRC-3 deficiency have improved insulin sensitivity and reduce glucose production and hepatic lipid accumulation [43]. Accordingly, SR18292, a synthetic molecule, directly promotes the PGC-1*α*/GCN5 interaction, which improves glucose homeostasis and insulin sensitivity and, as a consequence, ameliorates T2DM [235].

## 6. Conclusions

PGC-1*α* is a master regulator of mitochondrial biogenesis, oxidative phosphorylation, and mitochondrial antioxidant defense, and it is responsible for maintaining metabolic homeostasis. PGC-1*α* regulation is considered an adaptive mechanism to guarantee an adequate response to the metabolic requirements and also avoid the cytotoxic effects of the accumulation of ROS. Deregulation of PGC-1*α* expression may trigger metabolic disorders that can cause inflammatory process with altered redox control, contributing to the etiology and development of metabolic syndrome. Although much remains to be understood about the integrative molecular mechanisms involved in both the regulation and mode of action, PGC-1*α* modulation may be an interesting therapeutic target that may have significant benefits for a number of metabolic diseases.

## Figures and Tables

**Table 1:** Tissue-specific dysregulation of PGC-1*α* in metabolic syndrome.

Metabolic dysfunction	Tissue	PGC-1*α* status	References
Obesity	Adipose tissue	↓	[161, 167–170]
	Liver	↓	[176, 179]
	Skeletal muscle	↓	[176, 177]

Type 2 diabetes mellitus	Liver	↑	[182, 183]
	Kidney	↓	[191]
	Brain	↓	[192]
	Heart	↓	[193, 194]
	Retina	↓	[196]

Hepatic steatosis	Liver	↓	[58, 141, 204–209]

Cardiovascular diseases	Heart	↓	[89, 188, 219, 221]
	Endothelial cells	↓	[47, 60, 211, 212]
	Brain	↓	[206–208, 213–215]
